# Supplementary material for: Causes and consequences of sex-chromosome turnovers in Diptera
Source: Evol Lett. 2026 Mar 12;10(3):253–68. doi: 10.1093/evlett/qrag003 (PMC13231274; doi:10.1093/evlett/qrag003)
Supplement: qrag003_Supplemental_Files [file qrag003_supplemental_files.zip › Supplementary_Datasets.zip!/Supplementary_Datasets/Dataset_2_Graphical_representation_outgroups/Drosophilidae_Drosophila_innubila_chromosomes.pdf]

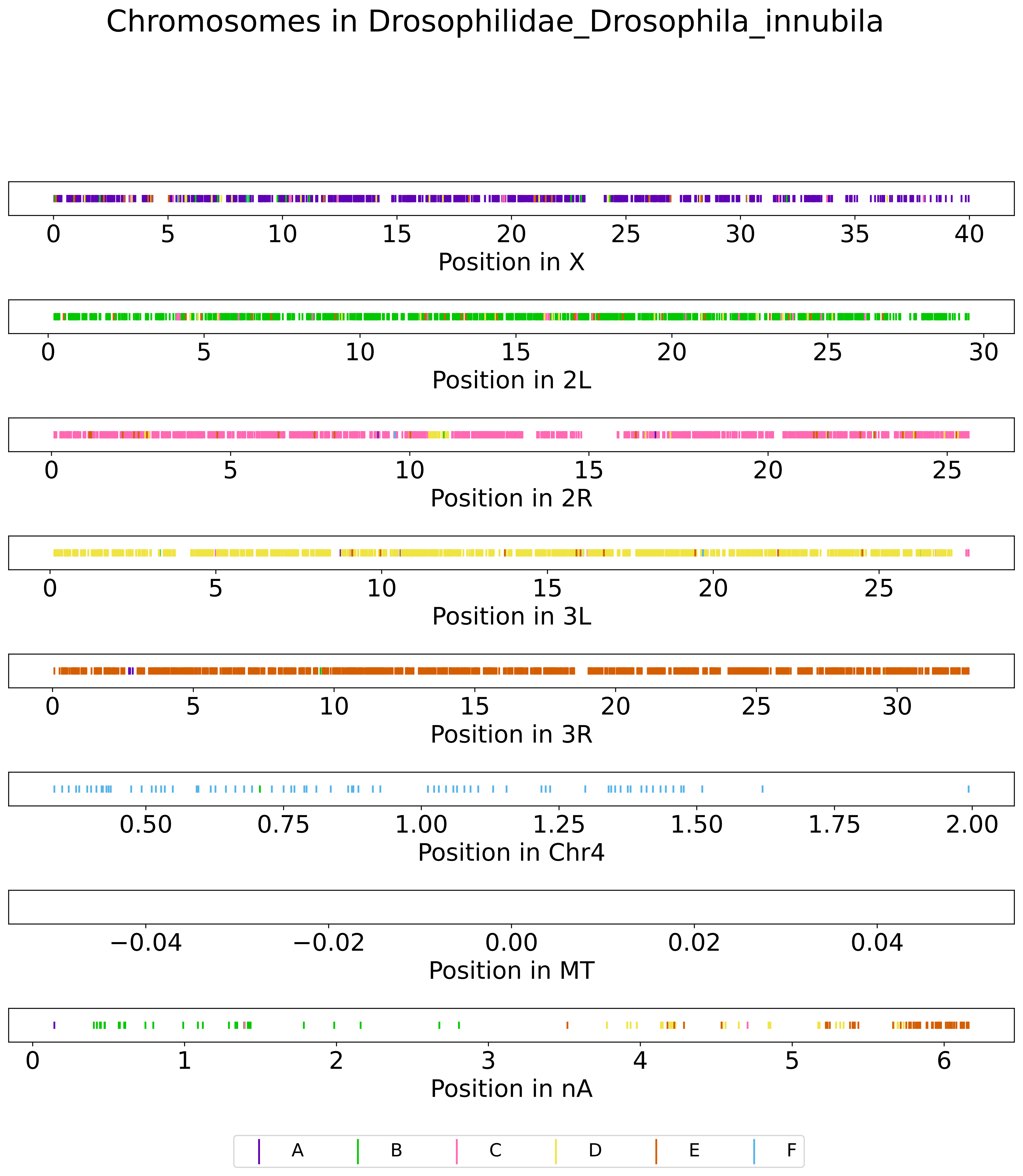
Chromosomes in Drosophilidae_Drosophila_innubila
0 5 10 15 20 25 30 35 40
Position in X
0 5 10 15 20 25 30
Position in 2L
0 5 10 15 20 25
Position in 2R
0 5 10 15 20 25
Position in 3L
0 5 10 15 20 25 30
Position in 3R
0.50 0.75 1.00 1.25 1.50 1.75 2.00
Position in Chr4
−0.04 −0.02 0.00 0.02 0.04
Position in MT
0 1 2 3 4 5 6
Position in nA
A B C D E F
